# Trying to find Safety, to make it Speakable, and to Mourn the Losses – Children’s Recovery from Domestic Abuse

**DOI:** 10.1007/s10896-024-00745-5

**Published:** 2024-10-04

**Authors:** Fiona Morrison

**Affiliations:** Childhood and Youth Studies Research Group, MHSES, University of Edinburgh, Edinburgh, UK

**Keywords:** Children and domestic abuse, Mothering and domestic abuse, Trauma, Recovery, Groupwork

## Abstract

**Purpose:**

This article addresses a gap in our understanding of children’s ‘recovery’ from domestic abuse. Whilst the impacts of domestic abuse on children have been well-documented and researched, their recovery from it has been under-theorized. By analyzing qualitative accounts on children’s participation in recovery programmes, the article uses Judith Herman’s trauma recovery model, to make the *how* of children’s recovery explicit.

**Methods:**

Conducted in Scotland, the research involved individual qualitative interviews with 14 children (aged 7-15 years) and their 14 mothers, who had completed Cedar, a 12-week therapeutic and peer support group programme. A co-produced approach to initial data analysis foregrounded children and mothers’ own priorities for children’s recovery and afforded findings greater rigor. These priorities were then further analyzed using Herman’s trauma recovery framework.

**Findings:**

Findings offer insight on the ways in which structures, resources, and values designed into the recovery programmes can mobilize children’s experiences of domestic abuse to help them find safety; make domestic abuse ‘speakable’; as well as provide space for children to mourn the losses resulting from domestic abuse. Findings further indicate the power of group-based interventions and the ways in which they can promote recovery through ideas of nurture and care.

**Conclusions:**

By using Herman’s theoretical lens for recovery, the article makes a new contribution to understandings of children’s recovery from domestic abuse. It identifies key factors that contribute to children’s ability to recover including: their sense of safety, the role of the mother–child relationship and significance of the child-father relationship.

The last thirty years have seen a rapid growth in knowledge about children and domestic abuse. Initially, children were largely absent in research and responses to domestic abuse. Historically, domestic abuse has been understood as an adult concern, a private issue, and a family matter (Kelly, 2003). Children’s needs, views, and experiences were not fully considered, if they were considered at all. While feminist scholarship challenged the treatment of the family as a ‘private sphere’, highlighting how this colluded with and made women vulnerable to men’s violence (e.g. Stark, 2007; Marcus 1994), child focused scholarship has brought children’s needs and perspectives to the fore in research, policy, and service responses to domestic abuse (e.g. Callaghan et al., 2018; Katz, 2016; Humphreys & Stanley, 2006; Jaffe et al., 2003; Mullender et al., 2002). These advancements in our knowledge about children and domestic abuse have been substantive and conceptual. Substantively evidencing the scale, ways, and the extent to which domestic abuse impacts children (e.g. Jaffe et al., 1990; Holt et al., 2008; Nation et al., 2023). Conceptually embracing and incorporating children’s views and accounts to expand understandings of what domestic abuse is and how it is experienced (e.g. Callaghan et al., 2018; Morrison, 2015; Øverlien, 2013; Mullender et al., 2002; McGee, 2000). Undoubtedly, this has marked major steps forward in understanding the issue of children and domestic abuse, however many questions are still unanswered. How can the impacts of domestic abuse be best ameliorated? What does recovery mean in this context? Can and how do children heal and recover? What role do mothers play in recovery? What about fathers? This paper looks to add to the knowledge base on children and domestic abuse by exploring these questions in relation to children and mothers’ ‘recovery journeys’. Drawing on interview data from a large-scale research project with children and mothers, the article analyzes their experiences of taking part in a therapeutic peer support and group work programme. By grounding the findings in Judith Herman’s (2015) established trauma recovery theory, the article contributes to the ongoing development of knowledge on children and domestic abuse, as well as the potential for applying trauma theory in recovery work with children.

The article begins by mapping the literature on children and domestic abuse, providing as summary of the existing evidence on children and domestic abuse, the group work programme itself and introducing Herman’s (2015) framework on trauma and recovery. This signposts the conceptual resources that underpin the later analysis. Next, the article outlines data collection methods for the larger-scale study are outlined, followed by a detailed discussion of the specific methods used for the data in this article, along with an overview of the participants involved. The methods section concludes with an account of key ethical issues and their impact on the collection, analysis and dissemination of findings. In the third section, the article presents the findings, integrating empirical data with Herman's trauma recovery theory, to expand understandings of how recovery occurs and the key factors that contribute to it. The fourth and final section draws together the significance of the findings, highlighting their role in enhancing our understanding of what matters to children in their recovery from domestic abuse. The article concludes by discussing the limitations and opportunities afforded by the approaches described and calls for further research that continues to advance children’s participation in research that is designed to influence their lives.

## Literature Review

Earlier research on children and domestic abuse was concerned with measuring the scale and impact of domestic abuse on children (e.g. Kitzmann et al. 2003; Wolfe et al., 2003; Jaffe et al. 1990). It focused on which children might develop problems and issues like the ‘cycle of violence’ that continue to be contentious (e.g. Haselschwerdt et al., 2019). There was a strong focus on the ‘protection of children’, but not really on addressing domestic abuse or on children’s recovery from it. In this context we see women becoming marginalised, and the rise in narratives like 'mothers’ failure to protect' in both policy and practice (Lapierre, 2008). While early child development focused research has been important, raising much needed awareness on the impact domestic abuse has on children, its impact on practice with children has been more limited.

Since the early 2000s, there has been a burgeoning of research underpinned by ideas of children’s rights and focusing on children’s own experiences of domestic abuse. This scholarship constructs children as rights holders, having their own views and needs (e.g. Morrison et al. 2020; Kelly & Mullender, 2000). This has acted to expand understandings of domestic abuse and of men’s violence against women and children. Children are less likely to be viewed as ‘hidden victims’, instead viewed as being intimately involved and affected by domestic abuse (e.g. Katz, 2022; Callaghan et al. 2018; Holt et al. 2008; Øverlien, 2014; Mullender et al., 2002; McGee, 2000). Through this, we see changes in the language used about children, reflecting the changing understandings of children in the context of domestic abuse – from witness to, exposed to, living with, experiencing. These changes reflect an evolving status that children occupy in understandings of domestic abuse, including in practice, policy, law, and research. Children are less likely to be positioned as passive victims, instead recognized as individuals with their own experiences, distinct needs, and unique expertise and understandings of domestic abuse.

Across feminist research and advocacy, we see children’s and women’s interests brought together in the context of domestic abuse. Liz Kelly (1994) constructed domestic abuse as a ‘double intentioned’ form of abuse – a combined attack on women and children. This ‘attack’ extends to the mother–child relationship, where as part of the abuse, direct and indirect attempts are made by the perpetrator to undermine the mother–child relationship. Forging an alliance between women and children was seen as a means to protect children. Morley and Mullender (1994:10) argued that while women’s and children’s interests may at times conflict, except where this was demonstrably and irrevocably the case, the most effective way to help children, was to understand what was happening to their mothers was seen to work in alliance with them. But women and children's needs and interests do conflict and cannot be assumed to synonymous or proxies for one another (e.g. Morrison and Houghton, 2023). The relationship between children and women can be simultaneously a source of strength and of vulnerability (e.g. Humphreys and Stanley 2006; Baly, 2010; Humphreys et al., 2015; Katz, 2016; Buchanan and Moulding 2021). So, it follows, that to support children’s recovery from domestic abuse efforts must be made to strengthen and restore the mother–child relationship. 

## Recovery Interventions for Children and Mothers when there is Domestic Abuse

The evidence base on how children are helped to recover from domestic abuse is limited, perhaps owing to the vulnerability of many of the interventions themselves. This makes the task of evaluation and researching them particularly challenging. There is often a lack of clarity as to the outcomes that domestic abuse orientated interventions for children can and should achieve and how these might be measured. Howarth et al (2015) argue for greater consensus on the outcomes, stressing the importance of ensuring these incorporate the views of the children and parents who use them. Arai et al. (2021) further highlight the importance of interventions recognizing the diversity of children’s experiences of domestic abuse, as well as their agency and hopes for their futures. Reporting on ideas of ‘readiness’ to participate in therapeutic interventions, Howarth et al (2019) recommend viewing parental and child readiness through an ecological lens, distinguishing between mothers’ awareness of the impact that domestic abuse has on their children and being able to shift their focus to addressing the needs of their children. Vikander and Källström (2024) report on the characteristics that children’s value in social workers when addressing domestic abuse. These range from being trustworthy, incorporating fun and enjoyment, and helping to validate children’s experiences of domestic abuse.

## Cedar and the Community Group Treatment Programme

Cedar has been established for over a decade in Scotland and as an intervention it is strongly rooted in the Community Group Treatment Programme (CGTP), a groupwork programme for children and mothers, pioneered in London, Ontario. Cedar continues to use the CGTP’s original structured 12-week curriculum and delivery, following much of their approach advocated in the 2000s (see Sharp et al., 2011, for an overview of these principles for delivery). Groups for children and mothers take place separately, concurrently and in parallel with one another. The focus of both children’s and mothers’ groups is children and domestic abuse. These groups explore children’s experiences, understandings, and feelings about domestic abuse, thereby helping mothers support their children’s recovery.

The CGTP model itself has long history, developed in the mid-1980s and its first evaluation reported by Jaffe et al. in 1986. Despite its longevity, research on the CGPT model is limited. Debbonaire (2007) reported positively on an iteration of the CGTP model in Sutton, UK. Children participating in the programme reported it helped them to learn about domestic abuse, feel less responsible for or alone in their experiences of domestic abuse, and increased their knowledge of safety planning. Children also reported being more able to deal with their feelings and talk their experiences. Sudermann et al. (2000) conducted a survey-based evaluation with children and mothers who completed the CGTP in London, Ontario. They similarly reported positively about children’s views on responsibility for abuse, their ability to talk about abuse, as well as mothers’ views that participation in group had helped their children. Sudermann et al. also point to areas where the intervention could develop. For example, mothers expressed a desire to know more about how their child was ‘doing in group’, and feelings that some children would have benefited from more support at the end of the programme. They further suggest the need to find ways to support children that find group settings challenging. Sharp et al. (2011) action research evaluation of the first piloting of Cedar in Scotland, echoed findings on the benefits brought about by the CGTP for children. They added new positive findings on aspects of strengthening the mother–child relationship and on families feeling more hopeful about their futures following their participation.

Together, these findings point to the benefits that interventions like Cedar can have for children in the context of domestic abuse. However less clear, is *how* interventions like Cedar contribute to and help children’s healing and recovery. How do they help children heal and recover? What are the ingredients for it? What are the things that hinder recovery? Herman’s (1992, 2015) work has been seminal in understanding survivors’ pathways to recovery, particularly from men’s violence against women and children. This article uses this trauma recovery framework as its conceptual basis to understand how children can recover in the context of domestic abuse. Her framework shows three key stages for recovery – establishing safety; reconstructing the trauma story through remembrance and mourning; and then reconnection between survivors and their wider community. Herman describes recovery as when the survivor can engage and find joy with everyday life (2015, p. 171).

There are two key differences between Herman’s articulation of her trauma recovery framework and the intervention Cedar: Herman’s focus is on adults’ recovery not children’s; and that focus concerns the recovery of the individual adult, not the mother–child dyad which Cedar explicitly engages with. Nonetheless, as the analysis shows, synergies exist between Cedar and Herman’s work.

## Methods

The research discussed here is one part of a more extensive UK-based research project, that examines innovations in social care for children and families in circumstances of domestic abuse. There are three substantive areas of inquiry within the project – child protection, criminal justice, and therapeutic interventions. This part of the research contributes to the third area of inquiry, it aims to explore how a model of group work supports children and their mothers to recover from domestic abuse.

Methods reported here include separate qualitative interviews with 14 children aged 7-15 years and their 14 mothers who had completed the Cedar programme. Interviews did not explicitly use the language of ‘recovery’, rather they traced and explored participants views of their wellbeing and safety across time. Interviews with children used a range of creative art-based methods (Tisdall et al., 2009) for example, physically mapping journeys to, through and from Cedar; sorting and ranking the ways in which Cedar helped; and inviting children to offer advice to other children, Quantitative measures on outcomes about children’s and mothers’ safety and wellbeing were also gathered with a larger study sample, these are reported elsewhere (Callaghan et al., forthcoming). Methods did not include observation of the groups themselves out of ethical concern for participants’ privacy and to limit the burden of the research on participants and the intervention itself.

## Participants and their Participation in Interviews

The research was conducted with participants spanning 4 women’s groups and 4 children’s groups. These groups were co-ordinated by 3 different group co-ordinators and supported by 3 different group co-facilitators. 8 of the child participants were girls and 6 were boys. Interviews took place within 2 months of completing Cedar, the majority were conducted by the author, and a minority by Friskney. The researchers met with families at the end of Cedar to introduce themselves and the research. Cedar then mediated the researchers access to families who wished to take part in the research. Not all families invited chose to take part in interviews. As access was mediated via Cedar, it is difficult to give definitive reasons for these decisions, reasons reported included: the service being unable to contact families; changes in families’ circumstances making participation difficult; and some families simply did not want to take part. All interviews with children were conducted face-to face, as were most interviews with mothers. 3 of the interviews with mothers were, at their request, telephone-based, enabling mothers to fit interviews into their schedules, including work and childcare. Most face-to-face interviews took place in families’ homes, three families asking that the interviews take place at Cedar. Interviews were semi-structured, and with permission were audio recorded and then transcribed. One child refused for their interview to be recorded, instead agreeing for the researcher to take notes during the interview and write it up afterwards. Despite reassurances of confidentiality and anonymity, the child was uncomfortable with being audio-recorded. Both children’s and mothers’ interviews focused on their journeys to, through, and after Cedar, asking what advice they would give to practitioners and policymakers on supporting children affected by domestic abuse.

## Approach to Data Analysis

Data was first analyzed by the author using Braun and Clarke’s (2006) reflective thematic analysis. As the author conducted majority of the interviews, most of the content was familiar to her in the initial coding stage. From this, themes were generated, then reviewed, and discussed with the wider research team. Themes were further refined and developed into a high-level analysis that participants of the research were then consulted on. Involving participants in the analysis was both a way to try and decenter power in the research process and to bring further rigor to the analysis (author; Coad & Evans, 2008). This was practically achieved by collaborating with an artist to translate the higher-level analysis into a visual map of families’ journeys through Cedar. Families who took part in the original interviews were then invited to two group-based workshops with the researchers to hear, provide feedback and ‘sense-check’ the developing analysis. A smaller sample took part in these sessions, 6 of the 14 children and 6 of the 14 mothers who had taken part in the original interviews. Reasons for not taking part in the analysis workshops ranged from: having other commitments, feeling they had ‘moved on’, and in one case difficulties arising from child contact arrangements meant that the mother felt participation in the workshops would be distressing for her child. During the workshops, participants’ responses to the analysis were recorded and incorporated into the analysis, resulting in two revised maps, one of children and one for mothers, these were then shared again and discussed with the child and adult participants. For more detail on the approach to coproducing analysis see Morrison (forthcoming).

Discussions during workshops and the resulting maps resonated with ideas of recovery and healing from domestic abuse. This prompted further analysis of the interview data using Herman’s trauma recovery framework as conceptual and analytical frames, which is what is discussed in this article.

## Ethics

Ethical approval for the study was given by the University of Stirling Ethics Committee. During the research informed consent and safeguarding and wellbeing were especially salient to the design and conducting the research. Informed consent was sought for all child and adult participants, covering key issues (e.g. accessible information, sharing why they have been invited to take part, confidentiality/anonymity, ongoing voluntary consent, contact details for questions/complaints, information on data sharing/archiving). All children under the age of 18 were asked to opt -in to taking part; children under 16 in Scotland (following the Age of Legal Capacity (Scotland) Act 1991) needed parental (mothers’) consent as well. Information and consent were adapted to suit participants' communication needs (e.g. accessible formats). The research had set up safeguarding protocols, including referral pathways for participants, and debriefing for the researchers. As part of informed consent, participants were informed of confidentiality limits, should there be concerns that someone is at risk of significant harm. A curated list of support organizations was given to child and adult participants during the research. All participants received a card and gift voucher to thank them for their participation in the research.

## Findings

The findings are organized around seven key themes: progressing towards recovery, setting the tone for recovery, safety, making the unspeakable speakable, a place to remember and mourn, the power of the group, and the role of the group leader in nurturing and care. These themes are underpinned by Herman’s broader key stages of recovery: establishing safety, reconstructing the trauma story through remembrance and mourning, and reconnecting survivors with their wider community.

## Progressing towards Recovery

Herman notes that progression through the stages of recovery is not linear (2015, p. 128), an idea that resonated particularly with mothers:

*‘It's not a straight path forward, it's sometimes, you go a little bit back, to go shooting forward.’ (Mother, 11)*

Mothers described times where they and their children had taken steps towards and steps away from recovery. Despite its messiness there was a sense amongst mothers that Cedar helped them develop the tools that enabled them and their children to continue moving towards recovery. Backward steps from recovery usually stemmed from the aftermath of domestic abuse—criminal and civil court proceedings, issues with housing and money, concerns about their children’s wellbeing. Thus, underscoring that while no longer living with the perpetrator of abuse, he and the aftermath of abuse continued to be keenly felt by mothers and children. Steps forward in recovery typically related to improvements in mothers’ relationships with their children, and major achievements like securing a new home, job, or reconnecting with family members following the ending of the relationship with the perpetrator of domestic abuse. Mothers also expressed a tacit feeling that a recovery meant rediscovering and reconnecting with themselves; this is illustrated in the following extract from an interview with a mother:

*Yeah. I'm definitely feeling, I don't really know how to describe it, I'm definitely feeling more myself. I can talk, and I'm feeling really good, just a few weeks after going into the group, I had noticed a huge, almost like confidence booster. (Mother 12)*

## Setting the Tone for Recovery

Herman’s model is underpinned by the idea that recovery takes place through relationships, linking the importance of restoring connection in recovery work. In Cedar, these connections and relationships are made amongst and between children, mothers, and group co-ordinators. Herman (2015, p. 111) argues the ‘survivor is the author and arbiter’ of their own recovery, thus approaches to recovery need to mirror and support the survivor’s empowerment. In Cedar, the authorship of recovery begins with referrals to Cedar being made directly by families rather than mediated by professionals. This means that families actively decide for themselves whether to take part. Careful consideration is given to how this decision is articulated by children. Before children’s participation, coordinators meet children along with their mothers and spend time with children on their own. Over several meetings, co-ordinators explore with children, whether they have a memory of abuse and if they can acknowledge that domestic abuse has taken place in their family. Any decision to take part in Cedar lies firmly with children and mothers, it is not part of a statutory child protection plan where ‘engagement in group’ is reported on. The importance of Cedar being separate from statutory provision is exemplified in an extract from an interview with a mother:

*And it was nice, because normally you talk to somebody, it goes back to a social worker, health visitor, police, wherever. Whereas it was good that it didn’t do that. You could literally just pour your entire guts out, and that was it. Nobody said anything, nobody did anything. (Mother, 3)*

First meetings between families and group co-ordinators helped set the tone for families’ relationships with Cedar, children and mothers described how valuable these were in making and building connections and trust. Children in particular described valuing co-ordinators who were positive, energetic, and fun. In the following extract, a child reflects on the importance of a sense of fun that was imbued in Cedar in enabling them to address domestic abuse:

*I think Cedar is great, it’s fun. [..] It’s serious but it does not feel serious. It’s disappointing that it has ended but it was great, and it helps. (Child, 6)*

Together, these qualities set the tone for families’ engagement with Cedar, helping them to feel optimistic about their participation in Cedar and optimistic for their own recoveries.

## Safety

The issue of safety is complex in the case of domestic abuse. Research showing that domestic abuse often continues and may intensify following separation, with children’s post separation contact arrangements becoming a focus for it (e.g. Spearman et al. 2023; Birchall & Choudhry, 2018). Herman (2015, p. 142) argues safety is necessary before recovery work can begin, but that no single event marks safety, rather transition to it is gradual, often occurring ‘in fits and starts’. Readiness for recovery is when a ‘rudimentary sense of safety or predictability in life’ has been achieved. For Cedar, this idea of predictability translates to a criterion whereby the mother and child are no longer living with the perpetrator of abuse. It means that while abuse may be ongoing or the aftermath still felt, children and mothers’ lives were not shaped by daily domination and control. This reflects Herman’s jarring but pragmatic view that the perpetrator of abuse cannot be relied on to achieve safety, so the survivor must achieve it for herself (2015, p 138.). As discussed earlier, Herman’s work is adult orientated, the idea that child survivors are responsible for their own safety is even less palatable than it is for adults. How can children (and at times mothers) be responsible for safety when their status and power is lesser and their vulnerabilities greater? How can recovery interventions like Cedar increase safety if they do not address the abuser and cause of harm directly?

Through interviews, children challenged conventional ideas on who can achieve safety and the ways it happens. Children described feeling safer because other aspects of their lives had flourished. In the extracts below children connect their sense of safety to their wider social worlds, and the strengthening of their relationships with their mother and siblings:

*Child 2: I definitely feel safer, because I've got my friend's, I've got my family, I’ve got my cat, I’ve got my pets.*

*Int: What kind of pets do you have? So, you've got a cat, and what else?*

*Child 2: I've got a guinea pig; I’ve got four fish. I’m lucky.*

*Int: Nice. See, before you came to Cedar, did you feel safe?*

*Child 13: Well, I didn't know about a lot. And then when I was in…when I was getting used to it, I felt safer, and safer to talk about my things.*

For many children, their mother’s participation in Cedar had acted to assuage their worries and concerns about her:

*Child 7: I thought that was good for my mum, because my mum had some problems as well and, yes, that was…I was happy that she went to her group, and I went to a group.*

Reassurance that through Cedar mothers were supported, meant children felt able to take part in the activities of Cedar and in their lives beyond it. Relieving children of worries about their mothers, helped recalibrate their relationships, which in turn helped children feel safer. This idea, that supporting mothers enables a rebalancing of the mother–child relationship and then enhances children’s safety and recovery, was echoed in interviews with mothers. Several mothers described Cedar helping them to be able to ‘get on with being a mother’ – a simple but profound utterance: simple in its restatement of a relationship; profound in acknowledging such an ordinary relationship had been absent. This connects with Herman’s indices of resolution, (2015, p. 171) where survivors can begin to focus on ordinary life, find pleasure in relationships, and they are able to be more interested in their future than the past.

However, achieving security or safety is not always ‘within children’s or mothers’ gift’. Several children described feelings of (un)safety and (in)security, despite no longer living with the daily domination and control of domestic abuse. At times, these feelings of feeling unsafe were difficult for the children to articulate and understand. The feelings did not always connect to specific events or people. For example, the unsettling uncertainty of fear’s origins are captured in the following:

*Child 11: I feel a bit safe, it’s like I don’t expect to not be safe, if you know…*

*Int: So, you feel a bit safe, but not completely safe?*

*Child 11: Mm-hmm.*

*Int: What do you think would have to change to make you feel safe, like what would…*

*Child 11: I’m not sure.*

*Int: Is there anything that you…are you able to…or do you know what it is that makes you feel unsafe?*

*Child 11: I don’t know what makes me feel unsafe, I think it is just…can’t remember the word for it, but it’s something.*

While children struggled to articulate why they had underlying and residual senses of being unsafe and unsecure, it is understandable when we consider the circumstances that one child experienced as ‘normal life’ before she, her sister, and her mother fled from her father:

*He used to like slap her [my mother] and that, and like hurt her, and I’d have to go in front of my mum, but then if [my sister] went in front, she’d usually just get pushed past, and then I’d go in front and tell mum to go. And then like, once mum had done upstairs and locked her door … cause we had a room that had a lock inside, and that was like the chill room. So, we’d go in there, and we had like a fridge in there, we had food, we had water, and we had a TV in there, so we were pretty good, until he managed to sneakily get in when me and my sister were going to the toilet. (Child 9)*

This description of intervening to protect her mother and sister, and retreat to what was effectively a 'panic room' during attacks, was taken for granted by the child and her siblings. Given the everyday and catastrophic challenges that domestic abuse can present to children’s sense of safety, it would seem unlikely that all children or their mothers will have achieved a keen sense of safety before joining Cedar. So, it seems unlikely that an intervention like Cedar is ‘enough’ to entirely restore the rudimentary sense of safety that is necessary for all children’s recovery.

Of particular importance to this sense of safety was children’s past and ongoing relationships with their fathers. Mothers reported that unpredictable, unsafe contact between children and fathers undermined their child’s sense safety, effectively blocking recovery pathways for both children and mothers. As is well documented in other research (e.g. Spearman et al., 2023), domestic abuse permeates time, space, and relationships; is often neither over, nor confined to the past, so much so that children and mothers’ sense of safety can continue to be undermined far beyond the duration of adults’ relationship together.

## Making the Unspeakable Speakable

At its core, Herman’s (2015, p. 11) framework aims to help those who have experienced trauma find ways to ‘come closer to facing the unspeakable’. She argues that in responding to traumatic atrocities, people try to ‘banish them from their consciousness’, resulting in these atrocities becoming ‘unspeakable’. While this desire to ‘bury the truth’ is powerful, efforts to deny atrocities are often fruitless, and lead to other forms of harm. Rather, Herman suggests we must remember and talk about the unspeakable events; to restore the social order they have upturned and allow individual victims begin to heal. In Cedar, children and mothers are supported to develop a language for domestic abuse, helping to surface and acknowledge the harm caused to children, and to children’s and mothers’ relationships with one another. In the extract below, a child reflects on how participating in Cedar’s activities supported him to articulate his feelings, which acted in a way to free him from them:

*Child 10: I lik[ed] how it helped me to do, say, talk about all my feelings that I had, and how I couldn't let them go [before] because they [my feelings] didn't let me, and then I just…they just really went.*

Creating opportunities for making the unspeakable – children’s feelings about and experiences of abuse—speakable can be foundational to their recovery from domestic abuse. Its potency echoes in research with children that finds feelings of shame, stigma and fear emanate from their experiences of domestic abuse (e.g. Mullender et al., 2002); and in research with women that finds they may underestimate children’s knowledge, memory and at times the impact of domestic abuse on their children’s lives (e.g. Edleson 1999). By making the atrocities of domestic abuse speakable for children, Cedar offers a way to begin to repair the damage done. These positive impacts, brought about by making domestic abuse speakable between children and mothers, and the effects on their relationships together, are reflected in the following interview extracts with two mothers:

*[…] It’s opened up doors for us to talk openly…because I always wanted to protect her and she always wanted to protect me, and we didn’t want to talk about anything, so it’s opened up gates that we can communicate together about anything that’s happened in the past, that you don’t have to worry about upsetting me, and I don’t have to worry about upsetting her. I let her say whatever she wants, and then we’ll talk about it, and then we’ll come to some conclusion, whatever it is. (Mother 14)*

*So, it was something that we could, because obviously we were both suffering. I kind of just shove all my stuff to the back of my mind, to deal with the kids first. So, it was nice that, it was something we could both do, and it would help her sort of, and me, be able to talk again. Because we used to be able to talk, and then the last few years, it's kind of, we drifted apart. (Mother 1)*

The majority of children expressed similar findings to those of their mothers about being more able to talk openly with them about domestic abuse. However, there was a significant minority of children who were more ambivalent about their ability to talk with their mothers. This is illustrated in the following extract from an interview with a child:

*Interviewer: Yeah. And what about you and your mum, do you ever talk about what happened, or do you not talk about it?*

*Child 13:I don’t really like to talk about it, ‘cause I don’t want a big issue again. Because every time I talk about something, mum thinks it’s a massive issue so then she goes and tells everyone*

In this case, the mother's response to her child discussing domestic abuse, before participating in Cedar, continued to inhibit the child's ability to speak about it with her mother. This offers insight into how domestic abuse and reactions to children talking about it can have lasting effects on the mother–child relationship, and on children's recovery. While a parallel model like Cedar enables children and mothers to develop a language about domestic abuse and to practice this amongst peers, its success in making domestic abuse speakable amongst children and mothers’ risks being more tenuous. Finding ways to bridge more direct communication between children and mothers might make domestic abuse more speakable in some families, as well as potentially offering greater benefits to the mother–child relationship, repairing the damage done and aiding in children's recovery.

## A Place to Remember and Mourn

Viewed through Herman’s recovery model, the work of Cedar is situated in the remembrance and mourning stage of recovery (2015, p. 176). Through its groupwork curriculum, children and mothers are encouraged to articulate, and then reframe their story of domestic abuse, acknowledging the impacts it had. This remembrance work helps normalize survivors’ responses to the atrocities they have experienced, affording language for the feelings and responses they have provoked. The goal of this recovery is the *integration*, not the *exorcism* of trauma, with survivors finding a restorative power in the ‘truth-telling’ about their experiences (p. 147).

### Mourning the Losses

There are many losses—imagined and real, material and abstract—that often go unrecognized in childhood experiences of domestic abuse: the loss of idealized versions of a mother, father and family; the loss of a home, pets, and belongings; the loss of a school, its familiar routines, rhythms and rules; the loss of a community with its friends, neighbors and relationships; and the loss of a particular future. Mourning such loss, asserts Herman (2015, p. 143) should be embraced as an act of courage and pride rather than of humiliation. In this way, a survivor (here a child) ‘can discover her indestructible inner life and take control of her own recovery’ (p. 153). Acts of remembrance and mourning affirms the child’s worth. Her trauma story becomes a new one, no longer seeped in feelings of shame and humiliation, but in feelings of dignity and virtue. The integration of trauma is never complete, life events invariably awaken and resurrect it, but the memory of trauma and the intensity of its losses fade (p. 158). Providing opportunities to talk about these experiences and begin to mourn was a vital role for the group work curriculum of Cedar. This extract from an interview with a child illustrates how Cedar helps achieve this:

*I would explain it (Cedar) like it was a thing that helps you with all your worries, and when you get sad about something, you just come to Cedar and talk about it… they really help when I’m sad about stuff, and I just talk to them about it and then they make me better when I'm sad. (Child, 8)*

### Reconstructing the Trauma Story

Mothers’ also spoke powerfully about the positive impact of being able to reconstruct their experiences of domestic abuse. In the following extract, one mother describes how participating in Cedar helped to change her view of herself, helping her to discover her own strength, and begin to restore her sense of self:

*I wouldn’t be where I’d be today if I hadn’t done Cedar, because I’d still be blaming myself, I would still be going back to the past the whole time, I wouldn’t be able to deal with talking about the past, I wouldn’t feel comfortable talking about the past, I wouldn’t have been able to help anybody else, because I’d have been back…never mind six years away from it, I’d have been back at six months. And that’s where I would have stayed, that’s where my mind would have stayed, and it [me] just…it helped me grow into a new sort of, if you like, or go back to the real [me]. (Mother 13)*

Children described this idea of reconstructing the trauma story differently from their mothers. They regularly described Cedar as helping them understand responsibility for abuse. But this was complicated and perhaps for children, the mourning stage is longer or runs at a different pace to adults. For some children, the reconstruction was not yet complete or simply different to that of mothers. For children, issues of responsibility for abuse and its aftermath were not always neatly resolved. Some children continued to feel responsible for abuse. Some were still confused and angry about the considerable impact that abuse, and their parents’ separation had on their lives. Some were still upset and angry that their mother had not left earlier. The residual feelings that children may have, are exemplified in the following quotation from a mother:

*If it can make you…because obviously there’s a lot of blame stuff in there with Cedar, and who is actually to blame, and obviously my son still…he doesn’t blame me anymore, but he still…there’s still that thing that he can’t let go of like, why didn’t you just leave? (Mother 3)*

Fathers — the ideal and the reality – were a considerable source of mourning and a persistent challenge to children’s reconstruction of the trauma story. The following extract from a child, highlights ways in which fathers can be a loss for children:

*Int: What were the kinds of things you were worried about, can you remember?*

*Child 1: Mostly my dad.*

*Int: What sorts of things about your dad?*

*Child 1: Just that he didn’t live with me anymore.*

*Int: So, was it that you were missing him, or was it…?*

*Child 1: Yes, I was missing him a lot.*

While some children’s feelings about fathers were ambivalent, these relationships, the presence and absence of them, were a constant in children’s narratives. Children found their fathers’ behavior and their subsequent feelings towards them confusing and therefore difficult to reconstruct or integrate. This is illustrated in the following extract where the child tries to make sense of her father’s abusive behavior towards her:

*Child 11: Really, my dad didn't like me, and then, but he did actually like me, but he doesn’t live with us anymore.*

In the context of domestic abuse, fathers are often framed as source of risk and harm that needs to be managed. This is understandable, and the risks and harms to children and women do need to be acknowledged and addressed. However, children’s narratives about fathers also underline the importance of recovery work engaging with the complexity of children’s feelings, and their relationships with fathers – past, present, and future – so that they can be integrated. In the extract below, another child reflects on how his feelings about his father remain unresolved and his struggles with this:

*Child 6: Mostly [we] talked about hurting and feelings and…mostly hurting and feelings, [..] And then I really missed it [Cedar] then, because I did have a little bit more to talk about, but then I talked about it to my mum sometimes, when sometimes I'm sad about my dad.*

Cedar helped children to air these feelings about their fathers, to mourn them and their relationships with them. However, the depth of these losses can be seismic and if they go on unresolved, they can act to complicate recovery from domestic abuse. Children continued to report that talking about fathers, both absent and present, as hugely difficult, perhaps indicating that children were not yet able to move beyond mourning.

The intricacy of emotions that can be provoked in children is illustrated in the following extract from interviews:

*Int: Is there anything that you're still worried about, or feel sad about?*

*Child 1: Still sad about my daddy.*

*Int: What is it you're sad about?*

*Child 1: Sad, I don’t get to see him, and my sister does get to see hers.*

*Int: Yes, you said that. When did you last see him, can you remember?*

*Child 1: It’s about when I was four or five […]. I'm jealous because my sister gets to see her dad. I said that yesterday morning, I think.*

Jealousy is a complex emotion to express and is one that is often not encouraged nor tolerated. Being able to discuss this openly with their mother was a significant step. It shows how interventions like Cedar can help children to mourn by supporting their communication and the unravelling of complicated feelings. Providing a vivid example of how Herman’s recovery framework and Cedar offer ways to reconnect victims with their experiences, to mourn losses and begin to integrate them.

## The Power of the Group

Herman asserts that trauma acts to dehumanize but in recovery the group acts to exalt, ameliorating survivors’ feelings of isolation, stigma, and shame (2015, p.173). However, for the group to be successful, its primary therapeutic tasks must be congruent with its members’ stage of recovery, otherwise the group risks being ineffective, and even harmful (pp.173–190). A clear understanding of the therapeutic task, as well as a structure and rhythm for the group protects its members from being overwhelmed by trauma. Cedar’s curriculum, with the rituals and routines of the weekly group meetings, offered structure and predictability to children and mothers. The beginning and ending of meetings were signaled by the opening and closing of a treasure chest, symbolizing the care and confidentiality members could expect for, and afford to, testimonies shared during group.

Herman (2015, p. 174) contends that through groups, survivors help each other bear the terror and confusion of traumatic memories, as well as the pain of grief and losses. The group provides a place for, and affords solemnity to its members’ losses, providing opportunity for them to grieve, to honour the past and imagine futures with new ideas and feelings of hope. Herman describes a mirroring that takes place when recovery groups form, by extending herself to other group members, the survivor becomes able to receive the ‘gifts that others give.’ The tolerance and compassion that she offers to other members of the group rebound on her. There is a sense of collective empowerment when survivors see each other as peers and equals, simultaneously in need of help, and able to help one another. These ideas are illustrated beautifully in the following extracts from children, where they reflect on the strength, they found in knowing they were not isolated in their experiences of abuse, and the contribution this made to addressing their own feelings of isolation, stigma, and shame:

*Child 3: ‘I am not alone. I felt when I went to Cedar it was, yeah, I felt safe, I felt secure.’*

*Int: What did you think about being with other kids who’d had the same sorts of things happen in their family?*

*Child 7: I felt like I wasn’t the only one.*

*Interviewer: Yeah.*

*Child 7: I felt better.*

According to Herman, the resources of the group are drawn upon and shared, resulting in the group having a greater capacity to bear and integrate traumatic experiences than any one individual has (p. 174). In the following extracts, mothers' expand on this describing how the group helped them to both bear and find resolution for their trauma:

*That was probably one of the hardest parts of going through Cedar. Was having to re-live things and it never went unresolved, if that makes sense? Like, if there was something that came up, we spoke about it and it was, sort of, put to bed almost, before the end of the session. I never left thinking, I’ve not dealt with something, or I should have spoken about that. (Mother 4)*

Herman (2015, p. 186) argues that integrative work on trauma happens towards the end of the group, with endings formalized with rituals of farewell. These were embedded in Cedar with celebrations, affirmations, and gifts, marking the achievements of children and mothers:

*That gift bag, well you don't expect that stuff at the end, so we got all they bits. And that was really nice, because we weren’t expecting anything. It was great, because that just gave you that extra wee buzz before you left, like, achievement. (Mother 10)*

For many, endings of group were difficult, some feeling it ended too quickly. Women made relationships with other women and were able to sustain support for one another out of group. However, this was harder for children to do independently. This highlights the need to think carefully about recovery for children following an intensive intervention, particularly if the pace between recovery for mothers and children diverges, and children’s own support networks are more limited and reliant on their mothers.

## The Power of Group Leader to Nurture and Care

The remembrance and mourning stage involves sharing experiences of past, not current, trauma. This sharing is facilitated by a group co-ordinator. As Herman notes, this is an emotionally demanding role in that it models how to hear trauma stories but not be overwhelmed by them. In Cedar this was demonstrated by the co-ordinator holding boundaries for the group, for example by repeating established rituals (such as the opening and closing of the treasure chest); ensuring that everyone’s voice is heard (p.180); and keeping the meeting activities to time – practical actions that, in their outward demonstration of care, nurture the safety that enabled mothers and children to participate in emotionally demanding sharing. In Cedar, nurturing safety and demonstrating care was not limited to the time-bounded group meetings but included the manner in which assessments were carried out; arranging transportation for families to get to group meetings; ensuring favorite snacks were available; and distributing affirmation cards that became children’s cherished objects used to remember and take pride in their work at Cedar. One mother identified with the unremarkable appearance of such care and valued it the more:

*From the minute you go through that door, at the beginning of group, before your bum’s even touched the seat, someone’s offering you a cup of tea. Now, for a lot of people, you don’t get that at home. It’s you that’s making that cup of tea and it’s you that has to get up and do something for yourself. So, for those two hours, yeah, two hours, we were in group, you were looked after. (Mother 7)*

That experience of being looked after, of being cared for, of your wellbeing prioritized by another person – the fact that this is notable is a poignant reminder of what families are recovering from.

## Discussion

This article sought to advance understandings of children's recovery from domestic abuse by integrating empirical data with Herman's trauma recovery theory. In doing so, it provides new insights on an under-researched area, emphasizing how theoretical frameworks can help deepen analysis of children’s recovery processes. By grounding the findings in established trauma theory, the article contributes to the ongoing development of this theory in the context of children and domestic abuse and demonstrates its potential for application in recovery work with children.

Before discussing the detail of recovery work, it is important to restate how fundamental ideas of nurture and care were to children and mothers’ feelings towards Cedar and viewing it as an intervention that improved their lives. The article highlights how nurture and care, as enacted by the group-coordinator, laid the ground for recovery. These were not just ‘add ons’ that can be dispensed with, nor should their impact be underestimated. By offering survivors choice and acting in ways that showed they valued survivors’ inherent worth, children and mothers were able to participate in and sustain therapeutic work that is of a high intensity. This is highly skilled work, requiring group workers to navigate complex and difficult emotions in the aftermath of domestic abuse, all the while creating and maintaining an environment where survivors work together to bear the terror and confusion of traumatic memories and the pain of grief and losses.

Now, to consider what recovery means in the context of domestic abuse. The article explored the complexities of recovery, which requires a balancing of optimism for recovery and healing—where survivors can focus on ordinary life, find pleasure in relationships, and be more interested in their future than the past—with the reality that domestic abuse can be unbounded, crossing time and relationships. Domestic abuse does not end at the point of separation (e.g. Spearman et al., 2023), its aftermath can be enduring and far reaching (e.g. Katz, 2022; Callaghan et al. 2018; Holt et al. 2008; Øverlien, 2014; Mullender et al., 2002; McGee, 2000). Therefore, it may be questionable whether children’s recovery is possible in a context when trauma is often still ongoing. The analysis revealed how messy recovery can be and how deeply entwined it is with issues of safety and risk. Children’s sense of safety can be bolstered, when their social worlds are enlarged and relationships with their mothers, siblings and wider family strengthened. Their sense of safety can also be enhanced when they were relieved from worries about their mother. Interventions like Cedar achieve this, by helping to rebalance the mother–child relationship, freeing children up to focus on other aspects of their lives. However, for some children, safety was elusive, and a sense of insecurity continues to weigh heavily on them. Herman’s framework was developed with adults and perhaps the concept of ‘rudimentary sense of safety’ is more attainable for adults than it is for children. This suggests that, in practice – and in law and policy too — there needs to be greater recognition of the everyday and catastrophic erosion that domestic abuse and its aftermath continue to present to some children’s sense of safety. Without first finding ways to stem it, and restore children’s fundamental sense of safety, children’s recoveries risk becoming tenuous if not comprised altogether.

A key aspect for children’s recovery involves helping children develop a language for domestic abuse and a vocabulary for their emotions about it. This helped make children’s experiences and feelings about them speakable – with their peers, mothers and families. Doing so, supported children to address the feelings of shame, stigma and fear that often characterize childhood experiences of domestic abuse (e.g. Mullender et al., 2002). Herman’s framework is rooted in ideas that recovery takes place in relationships. These echo and fit comfortably with other feminist scholarship that places prominence on the mother–child relationship, and confidence in it, to protect and support children recover from domestic abuse. Findings discussed here showed that this prominence and confidence is well founded for many children, but not for all. Just as relationships between children and women can be sources of strength, they can also be sources of vulnerability for children’s recovery (e.g. Humphreys and Stanley 2006; Baly, 2010; Katz, 2016; Buchanan and Moulding 2021). For some children, there is a risk that the mother–child relationship is not sufficiently strong, or interventions do not sufficiently strengthen it. This means they are unable to maximize opportunities for or routes to recovery, connecting with Howarth et al’s (2019) ideas on ‘readiness’ for interventions. The article therefore calls for more critical consideration of if, how and what more interventions can do to strengthen these relationships. And if they cannot, there needs to be consideration of what alternative models of recovery might look like where the mother–child relationship is not relied upon as the main vehicle for children’s recovery.

By adopting frames of loss, grief and mourning to explore children’s recovery, the analysis enabled an expansive and broader perspective on children’s experiences, and in turn their needs in recovering from domestic abuse. In the context of domestic abuse, fathers are often reduced to discussions about risk, for example whether post-separation contact is safe or not. This focus underlies well-founded critiques about family law’s failure to protect children and women in post-separation parenting arrangements (e.g. Morrison, 2015; Birchall & Choudhry, 2018). However, children’s narratives emphasize the importance of thinking about risk and more. The ending of adult relationships in the context of domestic abuse is not the same as the ending of, or the changes it brings to, the child-father relationship. There is a need to expand the ways fathers are considered in the context of children’s recovery, recognizing these relationships as forms of loss that children may experience and the complexity of the feelings that children may have about this. If these losses are not attended to in recovery work, they risk complicating and stymying children’s ability to reconstruct their story about trauma and recover from domestic abuse.

## Conclusion

By analyzing children and mother’s accounts of ‘recovery’ through the lens of Herman’s model of trauma recovery, the article has made explicit *how* domestic abuse interventions can take up Herman’s challenge to ‘*reconnect the fragments, to reconstruct history, to make meaning of their present symptoms in the light of past events’* (Herman, 2015 p. 13). Using Cedar as a case study, the article illustrates how children can find safety, make domestic abuse speakable, and mourn their losses through carefully facilitated experiences of therapeutic group work. It also underscores the precarty of some children’s sense of safety and the importance of restoring it for children’s recovery to be possible.

Other contributions have been adopting new theoretical frames—loss, grief and mourning – to expand understandings of children’s experiences of domestic abuse. It has also highlighted the role of the mother–child relationship and the significance of the child-father relationship for children’s recovery. In doing so, the article raises important questions about the relational complexities and tensions inherent in the concept of children’s recovery from domestic abuse, emphasizing the need for renewed attention on children’s sense of safety and dynamics of *both* the mother–child and the child-father relationship in recovery work.

## Strengths and limitations

This study makes a valuable and in-depth contribution to the limited evidence base on children’s recovery from domestic abuse. By involving participants in the initial data analysis, the study foregrounds the priorities of children and mothers in the recovery process, thereby enhancing the rigor of the findings. However, the study's depth comes from its focus on a deliberately small sample, which may limit the representation of different groups or perspectives. The study did not include direct observations of the groups, a decision made to respect participants’ privacy and minimize the level of intrusion and the burden the research placed on both participants and the service. As a result, the study relies solely on participants’ reports on aspects such as the skills of the group coordinators or the impact of group composition on recovery. Participants were recruited from one domestic abuse support service that employs a specific model of support. This means the study may not capture variation recovery journeys resulting from differences in models of support. To address this gap, future research could involve a larger and more diverse sample, including participants from various support models. Ultimately, it is important to continue exploring ways to involve children in research that is designed to impact their lives positively.
